# Novel Ex Vivo Model to Examine the Mechanism and Relationship of Esophageal Microbiota and Disease

**DOI:** 10.3390/biomedicines9020142

**Published:** 2021-02-02

**Authors:** Samuel Cass, Catherine Hamilton, Aaron Miller, Daniel Jupiter, Kamil Khanipov, Adam Booth, Richard Pyles, Timothy Krill, Gabriel Reep, Ikenna Okereke

**Affiliations:** 1 Department of Surgery, University of Texas Medical Branch, Galveston, TX 77555, USA; shcass@utmb.edu; 2 Division of Cardiothoracic Surgery, University of Texas Medical Branch, Galveston, TX 77555, USA; cfhamilt@utmb.edu; 3 Division of Microbiology and Immunology, University of Texas Medical Branch, Galveston, TX 77555, USA; aamiller@utmb.edu (A.M.); rbpyles@utmb.edu (R.P.); 4 Department of Preventive Medicine and Population Health, University of Texas Medical Branch, Galveston, TX 77555, USA; dajupite@utmb.edu; 5 Department of Pharmacology and Toxicology, University of Texas Medical Branch, Galveston, TX 77555, USA; kakhanip@utmb.edu; 6 Department of Pathology, Beth Israel Deaconess Medical Center, Boston, MA 02215, USA; alboothmd@gmail.com; 7 Division of Gastroenterology, University of Texas Medical Branch, Galveston, TX 77555, USA; tskrill@utmb.edu (T.K.); glreep@utmb.edu (G.R.)

**Keywords:** Barrett’s esophagus, microbiome, ex vivo

## Abstract

Rates of esophageal cancer have increased over the last 40 years. Recent clinical research has identified correlations between the esophageal microbiome and disease. However, mechanisms of action have been difficult to elucidate performing human experimentation. We propose an ex vivo model, which mimics the esophagus and is ideal for mechanistic studies on the esophageal microbiome and resultant transcriptome. To determine the microbiome and transcriptome profile of the human distal esophagus, the microbiome was assessed in 74 patients and the transcriptome profile was assessed in 37 patients with and without Barrett’s esophagus. Thereafter, an ex vivo model of the esophagus was created using an air–liquid interfaced (ALI) design. This design created a sterile apical surface and a nutrient-rich basal surface. An epithelial layer was grown on the apical surface. A normal microbiome and Barrett’s microbiome was harvested and created from patients during endoscopic examination of the esophagus. There was a distinct microbiome in patients with Barrett’s esophagus. The ex vivo model was successfully created with a squamous epithelial layer on the apical surface of the ex vivo system. Using this ex vivo model, multiple normal esophageal and Barrett’s esophageal cell lines will be created and used for experimentation. Each microbiome will be inoculated onto the sterile apical surface of each cell line. The resultant microbiome and transcriptome profile on each surface will be measured and compared to results in the human esophagus to determine the mechanism of the microbiome interaction.

## 1. Introduction

Despite advancements in surgical management and chemoradiation protocols, esophageal adenocarcinoma (EAC) remains one of the most lethal gastrointestinal malignancies with an overall 5 year survival rate of 17% and a proclivity for detection at an advanced stage [1,2]. Intestinal metaplasia of the distal esophagus, known as Barrett’s esophagus (BE), can occur in patients with chronic inflammation of the distal esophagus and is a critical risk factor for development of EAC. Patients with BE are recommended to undergo increased surveillance screening with endoscopic examination and biopsy of the esophagus [3]. BE is a crude risk factor, however, and only 1 in 860 patients with BE ultimately develop EAC [4].

Over the last several years, multiple studies have identified differences in the microbial community within the esophagus in people with BE compared to people without BE [5,6]. The normal esophagus is characterized by colonization with a high prevalence of Firmicutes phyla, of which *Streptococcus* is a member, while the esophagus of people with BE has a much higher percentage of Gram negative anaerobes [7]. The different microbial communities suggest two possibilities. Either the altered microbial community changes the mucosa of the esophagus or the altered intraluminal environment of the esophagus in patients with BE changes the microbial community. Yang and colleagues have proposed that Gram negative organisms play an important role in esophageal carcinogenesis, due to the release of lipopolysaccharides (LPS), which activate the innate immune system and result in chronic inflammation [8]. Furthermore, LPS are implicated in the activation of the proinflammatory NF-KB pathway and the exacerbation of reflux through the inhibition of gastric emptying [9,10].

Although these previous studies have outlined the phenomenological differences between patients with and without esophageal disease, there has been minimal research determining the mechanism by which these changes in the microbial community occur. In this study we describe an ex vivo model for examining the mechanism by which host–microbe interactions occur within the esophagus. Using a monolayer of immortalized esophageal cells with an air–liquid interface, a normal microbiome and Barrett’s microbiome will be transplanted directly onto the epithelial cell layer. This model can be manipulated to examine the mechanism by which different microbial communities interact with the esophageal mucosa and the differences in transcription and metabolomics profiles. We will also describe the experiments we performed to characterize the microbiome and transcriptome profile of the human esophagus. This human data will serve as the basis by which we compare the results from the ex vivo experiments.

## 2. Materials and Methods

### 2.1. Esophageal Microbiome

#### 2.1.1. Study Participants

The first aspect of the experiment was to perform the esophageal microbiome analysis. After institutional review board approval was obtained, 74 patients were recruited into the study. Patient demographics are shown in Table 1. All participants were (1) patients undergoing surveillance endoscopy for a known history of Barrett’s esophagus or (2) patients for whom screening endoscopy was recommended or could be considered based on guidelines from the American College of Gastroenterology. Indications for screening included men or women with chronic symptoms (greater than 5 years) of gastroesophageal reflux disease (GERD) and two or more risk factors for Barrett’s esophagus or esophageal adenocarcinoma: Caucasian race, age ≥ 50 years, chronic GERD symptoms, current or prior history of smoking, central obesity as defined as a waist circumference greater than 88 cm, waist to hip ratio greater than 0.8, family history of Barrett’s esophagus or family history of esophageal adenocarcinoma [11]. GERD was defined as a condition in which the reflux of stomach contents into the esophagus led to symptoms of heartburn, regurgitation or other signs related to reflux.

Demographic data was not available for the first 5 patients in the Barrett’s group. The average age in the entire cohort was 60.2 years. The majority of patients were currently on proton pump inhibitor therapy at the time of endoscopy, including 97% of the Barrett’s group and 88% of the GERD without Barrett’s group. Ten patients had long-segment Barrett’s esophagus, defined as a length of Barrett’s esophagus of three centimeters or greater.

When comparing the Barrett’s group to the GERD without Barrett’s group, there were no significant differences in age, gender ratio, BMI, tobacco use, presence of a hiatal hernia, current use of proton pump inhibitors or dose of proton pump inhibitors.

#### 2.1.2. Clinical Characteristics

Following endoscopy, physical examination and scripted interviews, the presence of Barrett’s esophagus, age, gender, body mass index (BMI), ethnicity, presence of a hiatal hernia, smoking history and use/dose of proton pump inhibitors were recorded. For patients with Barrett’s esophagus, the presence of dysplasia and the length of the Barrett’s column were also recorded. Barrett’s esophagus was defined as the presence of intestinal metaplasia in the distal esophagus. The presence of Barrett’s esophagus was confirmed histologically in every patient included in our study.

#### 2.1.3. Endoscopy

Prior to its use, the endoscope was sterilized and placed in a sterile container. The endoscope was then removed from the sterile container and placed directly into the esophagus. During the endoscopy, biopsies of the esophagus were taken from (1) normal esophagus from the proximal third of the esophagus (NP) and (2) normal esophagus from the distal esophagus, within one centimeter of the gastroesophageal junction (ND). In patients with Barrett’s esophagus, a mucosal biopsy of normal esophagus was taken within one centimeter of the gastroesophageal junction and adjacent to the Barrett’s esophagus. A swab of the uvula was also obtained using a sterile swab and immediately before the endoscopy was begun.

#### 2.1.4. DNA Extraction

Mucosal swabs and tissue biopsies were placed into sterile Powerbead tubes preloaded with 0.1 mm glass beads (Qiagen, Hilden, Germany, Germantown, MD, USA) plus external lysis buffer in vitro diagnostic (200 µL, Roche Applied Science, Indianapolis, IN, USA). Tissues were homogenized at 30 Hz for 5 min using a Tissuelyser II homogenizer (Qiagen). Sample lysates were deposited into individual wells of 96 deep-well processing plates. DNA was subsequently extracted in high-throughput fashion using a MagNA Pure 96 instrument running a DNA and viral small volume-in vitro diagnostic extraction kit according to the manufacturer’s protocol (Roche). After extraction, a portion of the DNA was evaluated by ion torrent next generation sequencing or using the esophageal microbiome array (EMB). The remaining material was archived at −20 °C.

#### 2.1.5. Next Generation Sequencing

Sample sequencing was carried out using a fusion-PCR method. Briefly, fusion-primers were designed in accordance with the manufacturer’s guidelines (Ion Amplification Library Preparation–Fusion Method, Life Technologies, Carlsbad, CA, USA) using Ion Xpress Barcodes linked to 16S gene primer pairs targeting hypervariable regions 1–8 [12]. Each 25 µL PCR was carried out using: 12.5 µL iQ supermix^™^ (Bio-Rad, Hercules, CA, USA), 1 µL of both forward and reverse (5 µM) primers, 9.5 µL nuclease-free water and 1 µL of DNA template. A total of 3 biopools of DNA created by equimolar mixing of the first 5 patient samples were analyzed. Each biopool represented DNA from the uvula swab, the NP mucosal tissue or the ND mucosal tissue. The DNA biopools were then used as templates for creation of subsequent fusion 16S libraries. PCR was completed in a c1000 thermocycler (Bio-Rad) using the following parameters: Cycle (1), 95 °C, 3 min, Cycle (2), Step 1: 95 °C, 45 s; Step 2: Primer-specific annealing temps., 45 s; Step 3: 72 °C, 2 min, repeat 39× and Step 4: 72 °C, 7 min. PCR products were purified using Qiagen Qiaquick spin-columns and quantified using a spectrophotometer (Bio-Rad). PCR products were then diluted, mixed in equal proportion and sequenced on an Ion Torrent GeneStudio S5 System using Ion 520 sequencing kits together with 520 size chips following the manufacturer’s instructions (Life Technologies).

#### 2.1.6. Bioinformatics for Ion Torrent

After generation, sequencing reads were filtered for quality and binned according to the Ion Xpress barcode using Ion Torrent Suite software version 5.10.0. Sequencing reads in FASTQ format were further processed using web-based Galaxy software [13]. First, raw FASTQ files were normalized using the FASTQ groomer tool function. Next, each barcoded read was trimmed to remove the primer sequence and subsequently filtered to the expected size of the 16S gene target. After this level of processing, the sequence reads were concurrently compared to the SILVA 16S database using bowtie 2 software [14,15]. This yielded a call to species or genera level and the number of times each sequence matched the database (hit-rate). When multiple calls to a genus were made, the number of hits was added accordingly. These numbers were then converted to percentage of total to give an overall ratio of the sequenced sample.

#### 2.1.7. qPCR Evaluation by EMB

To construct the EMB, Ion Torrent data and information from the esophageal disease literature [16,17,18,19,20,21,22,23] were compiled to select the most commonly detected organisms from the uvula to the distal esophagus. Ultimately a list of 46 targets was created that collectively represented greater than 85 percent of the detected microbiota in the Ion Torrent sequencing datasets. Two control qPCR targets were added to address the human DNA (hGAPDH) and total bacterial genomic loads (total 16S), creating a 48 target panel that was constructed in a skirted 96-well plate format (ThermoFisher Scientific Inc., Waltham, MA, USA). The 48 target array was constructed in the 6 × 8 format allowing for evaluation of 2 samples per 96 well plate (Table 2). Each 25 µL PCR was carried out using: 12.5 µL iQ SYBR green supermix^™^ (Bio-Rad), 1 µL of each forward and reverse (5–10 µM) primer, 9.5 µL nuclease-free water and 1 µL of DNA template. qPCR was completed in a c1000 thermocycler equipped with a CFX^™^ reaction module (Bio-Rad) using the following parameters: Cycle (1), 95 °C, 3 min, Cycle (2), Step 1: 95 °C, 30 s, Step 2: annealing 60 °C, 30 s, extension 72 °C, 30 s repeat 39×, Step 3: 72 °C, 2 min and Step 4: Melt-curve 75–89 °C, 0.2 °C temperature increments with 5 s plate read time.

Fluorescent signal data was collected at the end of each annealing/extension step. Starting quantity values were extrapolated from standard curves of plasmids harboring the PCR targets previously confirmed by Sanger sequencing. Any organism that was below the limit of detection was categorized as not detected. Mathematical analyses were performed using Excel^™^ (Microsoft Corp., Redmond, WA, USA).

#### 2.1.8. Statistical Analysis

The detection or non-detection of each organism was recorded in every sample in every patient. Two-way Firth-penalized logistic regression was used to relate the detection status to a selected variable (e.g., Barrett’s esophagus vs. GERD without Barrett’s esophagus) separately for each organism at each location. Two-way Firth-penalized logistic regression was used instead of conventional logistic regression due to the extreme values of detection incidence near 0% or 100% in many cases. The graphs for each organism were likewise modeled per 2-way Firth-penalized logistic regression, relating detection status to an association between a group (e.g., Barrett’s esophagus vs. GERD without Barrett’s esophagus) and a location (uvula, NP and ND). The graphs illustrate a model-predicted probability of detection at each location. To determine the association of the length of the Barrett’s column with microbiota, the Firth logistic regression was used for detection, restricted to the Barrett’s esophagus group only, controlling for the covariates to determine the association between location and length of the Barrett’s column. Statistical analyses were performed using R statistical software (R Core Team, 2018, version 3.5.1). In all statistical tests, α = 0.05.

### 2.2. Transcriptome Profile

#### 2.2.1. Study Participants and Sample Collection

To evaluate the transcriptome profile, 37 patients with (*n* = 9) and without (*n* = 28) Barrett’s esophagus were included in the analysis. Similar demographics were obtained and are shown in Table 3. Mucosal biopsies from the ND were used for the experiments.

#### 2.2.2. RNA Isolation

RNA was extracted using the Qiagen RNAeasy Micro Kit (Qiagen, Hilden, Germany). Esophageal mucosal biopsies were collected and stored in RNAeasy RLT lysis buffer with 1% B-mercaptoethanol. Samples were homogenized using a Bead mill 24 (Thermo Fisher Scientific, Pittsburgh, PA, USA) at a speed of 5 m/second for 30 s. Lysate slurry was centrifugated down and the supernatant was collected for subsequent steps. To the lysate, 70% ethanol was added and was transferred to an RNAeasy spin column. Spin column was washed using Qiagen RW1 buffer, RPE buffer and 80% ethanol. Final RNA collection was eluted with RNAse-free water. The final concentration was determined using a Qubit Fluorometer (Invitrogen, Carlsbad, CA, USA).

#### 2.2.3. Transcriptome Analysis

Total RNA was isolated from the mucosal biopsies and the RNA Integrity number was determined. Multiplexed RNA-seq libraries were generated for each sample using the Smart-3SEQ protocol [24]. The libraries were sequenced on an Illumina NextSeq550 at an average depth of 4 million 75 base pair single end reads. The sequenced reads were trimmed and filtered based on adapter content and quality. The CLC Genomics Workbench 20.0 was used for the bioinformatical analysis of the RNA-Seq data. Filtered sequencing reads were locally aligned against the *Homo sapiens* (hg38) reference genome, with annotated genes and transcripts at minimum matching length and similarity fraction of 90%. Resulting gene counts were normalized, and differential expression analysis was performed on the complete list of genes and transcripts to evaluate the level and significance of mRNA expression changes [25]. All significantly altered genes underwent pathway analysis using the ingenuity pathway analysis to highlight molecular function, biological process and cellular component to assess the host’s response. Clinical metadata, transcriptome profiles and microbial abundance were analyzed to identify significant associations between features from multiple measurement types. Hierarchical all-against-all association testing for correlation among all pairs of variables was performed using HAIIA 0.8.17. The resulting associations will be visualized in a network using Cytoscape 3.8.0 [26].

### 2.3. Ex Vivo Model

#### 2.3.1. Epithelial Cell Culture

**Figure 1:**
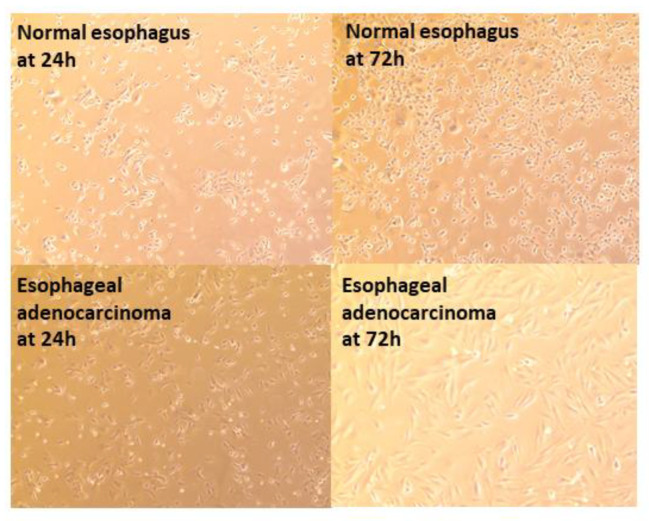
Normal esophagus and adenocarcinoma immortalized cell lines following thawing from liquid nitrogen at 24 and 72 h. Each image shows 4× magnification.

Primary and immortalized cell lines have been created previously by our lab using nasal and vaginal mucosa [27,28,29]. To create esophageal cell lines, immortalized cell lines were derived from human esophageal mucosal biopsies transformed with human papilloma virus E6E7. Primary and immortalized cell were cultured to form a monolayer of squamous cells representative of the esophageal epithelium. Normal esophagus and adenocarcinoma immortalized cell lines are shown at both 24 h and 72 h (Figure 1).

**Figure 2:**
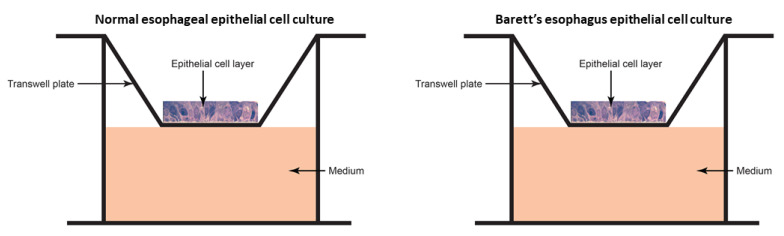
Ex vivo models for normal esophageal and Barrett’s esophagus epithelial cultures.

The monolayer cultures were cultivated in antibiotic-free keratinocyte serum-free medium (KSFM; Invitrogen, Carlsbad, CA, USA) with 50 micrograms/milliliter (ug/mL) bovine pituitary extract, 50 ug/mL bovine pituitary extract, 44.5 ug/mL calcium chloride and 0.2 mg/mL primocin (InvivoGen, San Diego, CA, USA). A monolayer model was created in the same fashion using epithelial cells of diseased Barrett’s esophagus. The monolayer was grown in a 33.6 square millimeter transwell cup (Fisher Scientific, Waltham, MA, USA). The ex vivo models are shown in Figure 2. The ex vivo model is ideal for esophageal microbiome testing for several reasons: (1) the apical surface of the epithelial squamous cell layer is exposed to air as in the esophagus, (2) the basal surface of the epithelial layer is in contact with a nutrient-rich medium and (3) the apical surface is sterile and can have various microbial communities transplanted for causative testing. It is important that the basal surface only is exposed to the nutrient-rich medium. This design of the model will allow the transplanted microbiomes to find nutrients in a similar fashion as in the human esophagus. This novel study design has allowed us to create experiments investigating potential mechanisms by which the esophageal microbiome causes disease. Though the ex vivo system is not an exact mimic, it does provide a good model for these studies.

#### 2.3.2. Microbiome Transplant

To prepare the microbiome transplant, mucosal biopsies of patients with and without Barrett’s esophagus have been collected. Within 30 min of collection, the microbiota were harvested from the samples and stored in lysate. Multiple normal samples were pooled to create the “normal microbiome”, and multiple Barrett’s samples were pooled to create the “Barrett’s microbiome”. We believe that sample pooling should yield an equivalent normal microbiome and Barrett’s microbiome that was seen in our preliminary experiments. In addition, the harvesting and processing of samples is identical to our preliminary experiments, which measured the normal and Barrett’s microbiomes. The lysate from each microbiome will be inoculated directly onto the epithelial layer of normal and Barrett’s EC cultures.

Our laboratory has previously performed this microbiome transplantation of the nasal microbiome. In that previous study, 20 different microbiome samples were transplanted onto nasal ex vivo models. The majority of the transplanted microbial communities mimicked the pretransplant in vivo communities [27]. Our technique of microbiome harvest and transplant in this study is exactly the same as our previously validated method.

## 3. Results

### 3.1. Esophageal Microbiome

#### 3.1.1. Demographics

A total of 74 total patients were enrolled in the study, including 34 patients in the Barrett’s group and 40 patients in the GERD without Barrett’s group.

#### 3.1.2. Microbiota Detection Patterns in Patients with and without Barrett’s Esophagus

The detection or non-detection of each organism was recorded in every sample in every patient. Two-way Firth-penalized logistic regression was used to relate the detection status to a selected variable (e.g., Barrett’s esophagus vs. GERD without Barrett’s esophagus) separately for each organism at each location. Two-way Firth-penalized logistic regression was used instead of conventional logistic regression due to the extreme values of detection incidence near 0% or 100% in many cases. The graphs for each organism were likewise modeled per 2-way Firth-penalized logistic regression, relating detection status to an association between a group (e.g., Barrett’s esophagus vs. GERD without Barrett’s esophagus) and a location (uvula, NP and ND). The graphs illustrate a model-predicted probability of detection at each location. To determine the association of the length of the Barrett’s column with microbiota, Firth logistic regression was used for detection, restricted to the Barrett’s esophagus group only, controlling for the covariates to determine the association between location and length of the Barrett’s column. Statistical analyses were performed using R statistical software (R Core Team, 2018, version 3.5.1). In all statistical tests, α = 0.05.

**Figure 3:**
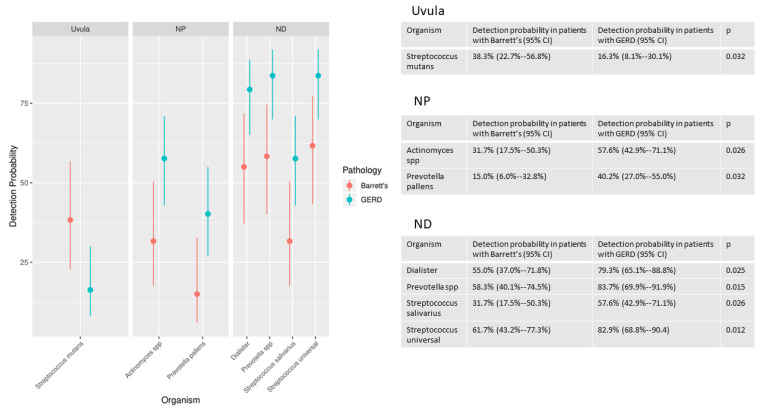
Probability of detection of organisms.

There were statistically significant differences in the likelihood of detection of multiple organisms in the Barrett’s group compared to the GERD without Barrett’s group. There were significant differences in likelihood of detection in one species (*Streptococcus mutans)* at the uvula, two genera or species (*Actinomyces, Prevotella pallens)* at the NP and four genera or species (*Dialister*, *Prevotella* unspecified, *Streptococcus salivarius* and *Streptococcus* unspecified) at the ND (Figure 3).

#### 3.1.3. Severity of Barrett’s Esophagus versus Microbiome Pattern

**Figure 4:**
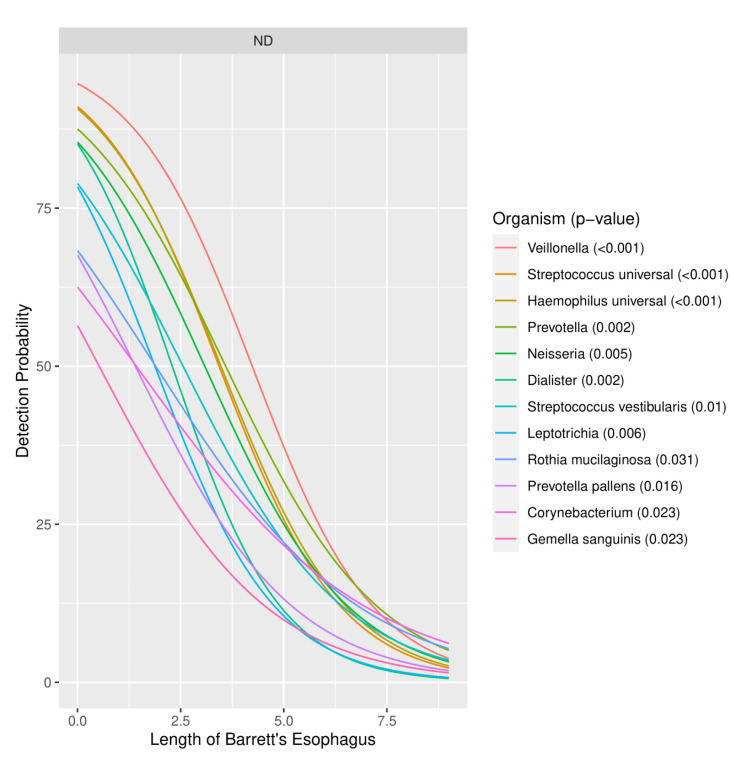
Probability of detection of organisms vs. length of Barrett’s esophagus.

There was a decreased likelihood of detection of multiple organisms as the length of the Barrett’s column increased. In particular, 10 different genera (*Corynebacterium*, *Dialister, Gemella, Haemophilus, Leptotrichia, Neisseria, Prevotella, Rothia, Streptococcus* and *Veillonella*) on the EMB array had a significantly decreased likelihood of detection as the length of the Barrett’s column increased (Figure 4). Given that 2-way Firth-penalized logistic regression was used, the curves show the probability of detection of an organism being detected for any length of Barrett’s esophagus. The only curves included in Figure 4 are for organisms for which length is a significant predictor at an α ≤ 0.05. 

### 3.2. Transcriptome Profile

#### 3.2.1. Demographics

Patient demographics are shown in Table 3. Average age was 61.7 years. Barrett’s esophagus was present in 24% (9/37) of patients. Average BMI was 30.4. No patients with Barrett’s esophagus had dysplasia. When analyzing patients with and without Barrett’s esophagus, the only demographic that was significantly different between the two groups was BMI.

#### 3.2.2. Cluster Analysis

**Figure 5:**
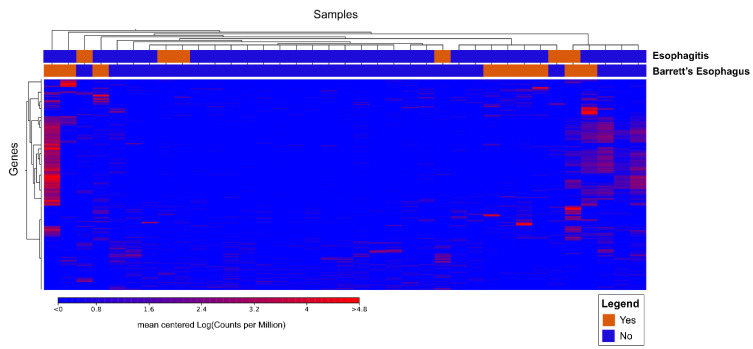
Cluster analysis data of 36 patients with and without Barrett’s esophagus.

There was a distinct transcriptome profile seen in patients with Barrett’s esophagus. Cluster analysis data is shown in Figure 5. There were a large number of genes that were differentially expressed when comparing the two groups.

Many of these differentially expressed genes are involved in cell motility, apoptosis, stress response and biological processes typified in highly replicative malignant tissues. A partial number of these genes is listed in Table 4. For a complete list, see Supplementary Table S1 containing all differentially expressed genes.

#### 3.2.3. Ingenuity Pathway Analysis

**Figure 6:**
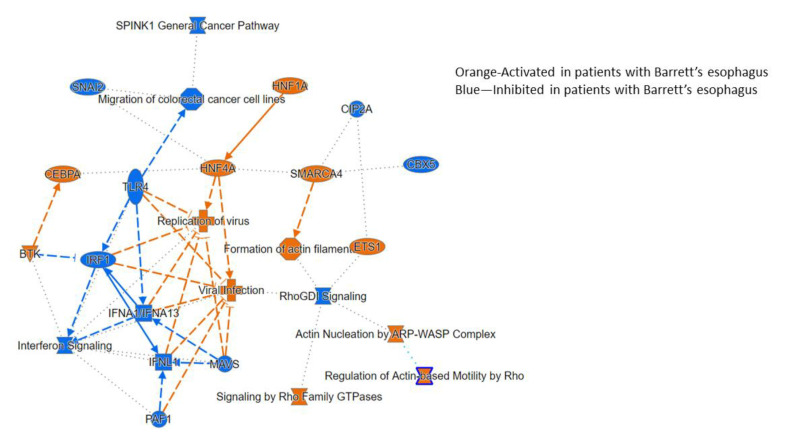
Ingenuity pathway analysis of processes related to Barrett’s transcriptome.

Ingenuity pathway analysis was used to map specific functions and biological processes that were related to differences in the transcriptome profile (Figure 6).

### 3.3. Ex Vivo Model

#### 3.3.1. Epithelial Cell Layer

**Figure 7:**
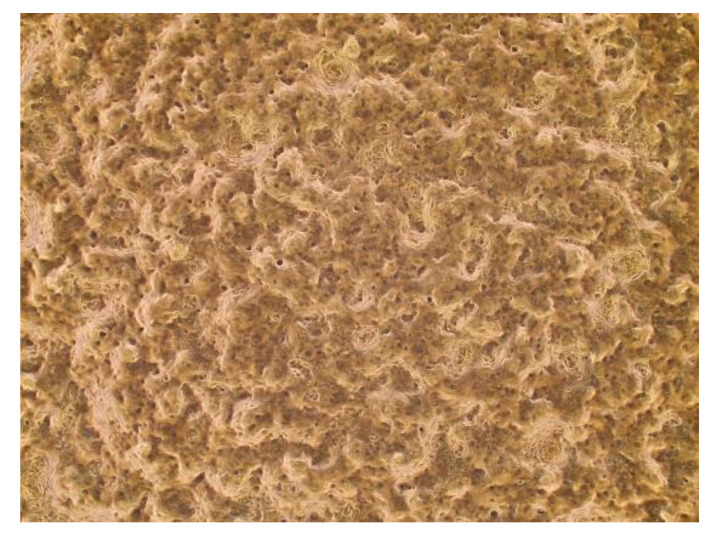
Histology of normal EC mucosal layer successfully grown on air-interfaced plate. The magnification scale is 4×.

An epithelial cell layer of squamous cells was successfully grown onto the ex vivo model (Figure 7). The microbiome transplants will be inoculated onto the apical surface of the cells. The resultant microbiome will be measured. 

#### 3.3.2. Transcriptome Analysis after Transplant

The supernatant in the ex vivo model will be collected. The resultant transcriptome profile will be measured.

## 4. Discussion

There is a dynamic relationship between a host and its surrounding microbial community. A disruption in this symbiotic relationship can lead to adverse consequences. Other organ systems have seen a disturbance in the microbiome result in disease and worsening health status [30,31,32]. *Helicobacter pylori* levels have been associated with GERD and esophageal cancer in some studies, but this trend has been inconsistent [33,34]. Bacteria can act as direct promoters of cancer progression, but can also shield the host from disease [35]. In an effort to elucidate details of the complex role of the microbiome in esophageal disease, we aimed to associate variations in the microbial community with esophageal disease. We further attempted to create an ex vivo model, which would allow for robust causative testing.

Our research suggests that there are organisms in the esophagus, which have a protective effect against esophageal disease. In our studies, we demonstrated a decreased frequency of detection of certain organisms in the presence of Barrett’s esophagus. We also showed that the detection of these organisms decreased as the length of Barrett’s esophagus increased. Both of these clinical conditions are risk factors for esophageal cancer [36,37,38]. As such, we postulated that similar bacterial trends might be associated with the development of esophageal cancer. Our studies also showed that there is a distinct transcription profile in patients with Barrett’s esophagus, with a large number of genes, which were differentially expressed. As described above, these genes regulate critical pathways of tumor biology, including cell motility, apoptosis and cellular stress responses.

There is a paucity of data in human experiments. Previous literature has used much more expensive and less efficient experiments on human tissue. This design is inefficient and may explain the relative lack of information on this important clinical dilemma. The benefit of an ex vivo model is that microbiome experiments can be performed repeatedly in a controlled environment. Additionally, this environment would not be subject to any interpatient variability, which likely exists in a large human cohort. Other ex vivo esophageal models have been created [39,40]. However, no previous literature has used an ex vivo esophageal model for microbiome studies. The ex vivo model is a good replication of the human esophagus, even if not an exact duplication and allows for control of the environment in sterile conditions. Our studies have shown that a sterile epithelial layer can be grown in an air-interfaced system. This design will allow for investigations on the relationship between mucosal cell function and its surrounding environment. We hypothesize that the microbiome will be one of the environmental factors, which affects the esophageal mucosa.

Our pathway analyses yielded potential mechanisms by differentially expressed genes may affect the esophagus. As an example, there were multiple differentially expressed genes in our study, which are involved in tumor suppression such as the EGR-related genes. This set of genes raises the possibility that inhibition of tumor suppression may be a mechanism by which the microbiome can increase the risk of esophageal cancer development. Future studies will be needed to examine specific genes/proteins, but these identified pathways may allow for treatment possibilities or diagnostic testing to identify patients at increased risk of cancer development.

With our design, we will be able to perform measurements on the resultant microbiome after manipulations on the ex vivo system. This system will allow us to understand whether the microbiome shifts as a result of the change in esophageal mucosa, versus the microbial shift causing the esophageal mucosa to change. This design allows for investigation of cellular and histologic changes, such as the proliferation rate and apoptosis. Additionally, this design will permit investigations into molecular changes caused by shifts in the esophageal microbiome.

One limitation of our ex vivo model is the possibility that the environment may not be a perfect surrogate for the human esophagus. In cell culture, epithelia may behave differently when culture with stromal cells than when cultured in a monolayer [41]. Our design has the added advantage that it is a multilayered epithelial surface, but further studies are needed to determine how well this system mimics the esophagus. Another limitation of our study is that we included patients who did and did not use proton pump inhibitor medications. However, the majority of patients in our study did use proton pump inhibitors. Additionally, a recent study by our investigative team showed that there were no differences in the esophageal microbiome in patients who did and did not use proton pump inhibitor medications [42].

## 5. Conclusions

Our experiments showed several key findings. There is a distinct microbiome in patients with Barrett’s esophagus. There is also a unique transcriptome profile in these patients, with a large number of genes, which are differentially expressed. Ultimately, experiments using this ex vivo model could create new surveillance regimens and therapeutic interventions. Probiotic supplements containing protective organisms could be administered to prevent esophageal disease. Surveillance strategies, which currently use histologic changes primarily, could include microbiome shifts to guide treatment. With a more comprehensive knowledge of the association of the microbiome and esophageal disease, we can better prevent and treat esophageal disease.

## Figures and Tables

**Table 1:** Esophageal microbiome cohort demographics *.

	Barrett’s Esophagus	GERD without Barrett’s	*p*-Value
N	34	40	
Male	62% (18/29)	50% (20/40)	0.32
Age, years (mean)	61.7 ± 10.7	59.0 ± 8.9	0.26
BMI (mean)	31.5 ± 8.7	31.1 ± 5.5	0.83
Hiatal hernia	52% (15/29)	35% (14/40)	0.16
Current smoker	24% (7/29)	20% (8/40)	0.61
Current PPI use	97% (28/29)	88% (35/40)	0.23
Mean PPI dose (milligrams)	46.2	37.0	0.11

* Demographic data was unavailable in 5 patients in the Barrett’s esophagus group.

**Table 2:** Esophageal microbiome array (EMB) qPCR array.

*Actinomyces*	*Lactobacillus*	*Rothia mucilaginosa*	*Campylobacter*	*Lautropia*	sal 337
*Campylobacter consius*	*Leptotrichia* unspecified	*Streptococcus anginosis*	*Campylobacter showae*	*Leptotrichia wadei*	*Streptococcus mutans*
*Capnocytophaga*	*Mycoplasma faucium*	*Streptococcus oralis*	*Corynebacterium*	*Neisseria*	*Streptococcus pneumoniae*
*Dialister*	*Porphyromonas endodontalis*	*Streptococcus salivarius*	*Filifactor alocis*	*Porphyromonas gingivalis*	*Streptococcus sanguinis*
*Fusobacterium nucleatum*	*Prevotella denticola*	*Streptococcus thermophilus*	*Fusobacterium periodonticum*	*Prevotella intermedia*	*Streptococcus vestibularis*
*Gemella sanguinis*	*Prevotella melaninogenica*	*Streptococcus*	*Haemophilus haemolyticus*	*Prevotella nigrescens*	*Veillonella*
*Haemophilus influenza*	*Prevotella oris*	*Veillonella atypica*	*Haemophilus parahaemolyticus*	*Prevotella pallens*	*Veillonella parvula*
*Haemophilus*	*Prevotella*	*Haemophilus parainfluenzae*	*Prevotella timonensis*	Total 16S	Human GAPDH

**Table 3:** Esophageal transcriptome profile cohort demographics.

	Barrett’s Esophagus Group	GERD without Barrett’s Group	*p*-Value
*N*	9	28	N/A
Age (years)	63.3	61.2	0.58
BMI	26.9	31.5	0.03
Hiatal hernia	56% (5/9)	32% (9/28)	0.21
Current smoker	22% (2/9)	25% (7/28)	0.99
Current PPI use	89% (8/9)	86% (24/28)	0.81
Mean PPI dose (milligrams)	37.8	36.4	0.87

**Table 4:** Partial list of genes differentially expressed in Barrett’s esophagus compared to normal.

Transcription Factor	Function
FosB	DNA binding
Early growth response protein 1 (EGR1)	Tumor suppressor
Early growth response protein 3 (EGR3)	Tumor suppressor
Nuclear receptor subfamily 4 group A member 1 (NR4A1)	Energy homeostasis
Cyclic AMP-dependent factor ATF-3 (ATF3)	Transcription repressor
Hepatocyte nuclear factor 4-alpha (HNF4A)	Transcription repressor
Ankyrin repeat and SAM domain-containing protein 4B (ANKS4B)	Epithelial brush differentiation
Galectin-4 (LGALS4)	Cell assembly
Apoptosis facilitator Bcl-2-like protein 14	BCL2L14
Cyclin-dependent kinase inhibitor 2A (CDKN2A)	Cell proliferation
Matrix metalloproteinase 7 (MMP7)	Cell degradation
E3 ubiquitin-protein ligase Mdm2 (MDM2)	Apoptosis
Regenerating islet-derived protein (REG)	Inflammation
Calpain 8 (CAPN8)	Cellular apoptosis
Defensin beta 103A (DEFB103A)	Regulation of cytokine production
Rho GTPase Activating Protein 26 (ARHGAP26)	Protein coding
Cell division cycle 25B (CDC25B)	Regulation of mitosis
G protein subunit beta-2 (GNB2)	Antiproliferative function

## Data Availability

The data presented in this study are available on request from the corresponding author. The data are not publicly available due to privacy concerns.

## Supplementary Materials

The following are available online at https://www.mdpi.com/2227-9059/9/2/142/s1, Table S1: List of differentially expressed genes in patients with Barrett’s esophagus.
